# Advanced age is not a predictor for cochlear implantation outcomes in adults with moderate to profound sensorineural hearing loss

**DOI:** 10.1016/j.bjorl.2025.101571

**Published:** 2025-03-21

**Authors:** Dayse Távora-Vieira, Andre Wedekind, Aanand Acharya, Jafri Kuthubutheen, Marcus Voola, Vinicius Cavalheri, Peter Friedland

**Affiliations:** a Fiona Stanley Fremantle Hospitals Group, Western Australia, Perth, Australia; b The University of Western Australia, Medical School, Division of Surgery, Perth, Australia; c Curtin University, School of Occupational Therapy, Social Work and Speech Pathology, Faculty of Health Sciences, Perth, Australia; d Sir Charles Gardner Hospital, Western Australia, Perth, Australia

**Keywords:** Cochlear implant, Age related hearing loss, Binaural hearing, Cochlear implant outcomes

## Abstract

**Objective:**

Hearing impairment is a common disability in elderly people. With an ageing population, it is expected that the number of people with age-related hearing loss will grow, and a proportion are likely to become Cochlear Implant (CI) candidates. The aim of this study is to investigate whether the age at which a person receives a CI has an impact on their objective speech understanding outcomes and subjective, self-reported outcomes within the first 12-months of CI use.

**Methods:**

242 CI recipients participated in this study. Participants were divided into 3 groups according to age at implantation: 72 younger adults (18–60 years); 109 older adults (60–75 years); and 65 elderly adults (>75-years). Benefit of CI use was assessed via speech understanding and patient-reported measures. Speech understanding in quiet was assessed via the Consonant-Nucleus-Consonant words test. Speech understanding in noise was assessed via the Bamford-Kowal-Bench Adaptive speech-in-noise test. Self-perceived hearing ability was assessed via the short from of the Speech, Spatial and Qualities of Hearing scale. Tinnitus reduction, if applicable, was assessed via the Tinnitus Reaction Questionnaire. All 4 assessments were completed at 4 intervals: preoperatively, 3-, 6-, and 12-months of CI use.

**Results:**

For the entire cohort over time, significant improvements were found for speech understanding in quiet (*p* < 0.0001), and self-perceived hearing ability (*p* < 0.0001). Between the three groups, there were no significant differences in any postoperative (post-Op) assessment for all study outcomes.

**Conclusion:**

Each age group had improved speech understanding and self-perceived hearing abilities after 12-months of CI use. No significant differences were found between groups at all post-Op test intervals. We therefore conclude that elderly people (>75-years) who are acceptable CI candidates should be offered the same opportunity to benefit from CI use as younger adult candidates.

**Level of evidence:**

Level 3.

## Highlights

• A CI improves speech understanding and subjective hearing results within the first 12-months.
• Outcomes were not dependant on age at implantation.
• Cochlear implants should be offered to all eligible patients.

## Introduction

Hearing loss is the fourth leading cause of disability worldwide and a growing public health concern, particularly as populations age.^1^ HL is associated with social isolation, reduced Quality of Life (QoL), depression, anxiety, and cognitive decline.^2^, ^3^, ^4^ Nearly 15% of individuals in the United States over the age of 12-years, and 66% of those over 70-years, have a bilateral hearing loss.^5^ For individuals with severe to profound HL who gain insufficient benefit from hearing aids, Cochlear Implants (CIs) are the preferred treatment. However, it is estimated that fewer than 10% of eligible CI candidates receive them,^6^ with advanced age cited as a significant barrier to implantation.^7^

Elderly individuals often face concerns about the surgical risks and rehabilitation demands associated with CIs. However, multiple studies consistently show that CI surgery is well tolerated in older population, with rates of major complications (such as meningitis, facial nerve paralysis, device failure, flap dehiscence, wound breakdown, implant migration) and minor complications (temporary vertigo, temporary facial nerve paralysis, facial nerve stimulation) comparable to younger CI recipients.^8^, ^9^ This indicates that advanced age is not a contraindication for CI candidacy, supporting individualised assessments for implantation.

CI outcomes vary widely among adult recipients, influenced by factors such as preoperative hearing thresholds and the duration of HL.^10^ Few studies suggested that age at implantation negatively affects outcomesm for instance, Blamey et al., did a retrospective analysis of the speech understanding in quiet in 808 adult CI users with post lingual deafness and found that speech outcomes negatively correlated with both duration of deafness and age at implantation.^11^ Subsequent studies supported the results of Blamey et al. (1996), showing that older adults perform poorer with their CI compared to younger adults.^12^, ^13^, ^14^ Nevertheless, almost two decades later, Blamey et al. repeated their analysis, this time with an even larger cohort (n = 2251), and found that duration of deafness and age of implantation were less important factors, when compared to their earlier study.^15^ Improvements in surgical technique, sound processing strategies, and the broadening of candidacy criteria to include people with better residual hearing have improved CI outcomes, with more recent research showing that age is no longer a significant determinant of speech intelligibility and Quality of Life (QoL) improvement.^9^, ^16^, ^17^, ^18^, ^19^, ^20^ Wong, Dillon, and Hast, found no significant decline in performance related to aging, especially in quiet conditions. However, noise conditions presented greater challenges for older adults in some cases, as observed by Hast (2015) and Zwolen (2014).

Patient-reported outcome measures are also an important tool to demonstrate the benefit (or lack thereof) of CI use. Studies have found that Quality of Life (QoL) improves significantly with CI use in elderly recipients, with age at implantation not impacting the results,^8^, ^21^ with the degree of QoL improvement often correlating with improvements in speech intelligibility.^22^ Furthermore, tinnitus intrusion may be significantly reduced with CI use^21^ with improvement being independent of age, when comparing cohorts over 70 years old with those aged under 70 years.^19^

As life expectancy increases, it is anticipated that the number of CI candidates will also increase with the average age at implantation potentially being older. While the current literature have shown that age is not a significant determinant of success, most studies have focused on broad comparisons between younger and older adults, often finding no significant differences in speech understanding outcomes. However, challenges like speech perception in noisy environments and tinnitus reduction remain underexplored, especially within specific subgroups of older adults, such as those aged over 75. Furthermore, while patient-reported outcomes like self-perceived hearing ability are critical to understanding the overall impact of cochlear implantation, they have received less attention compared to objective measures. This study seeks to address these gaps by examining outcomes across three distinct age groups (18–<60 years, 60–<75 years, > 75-years), focusing on both objective and subjective measures over the first 12 months of cochlear implant use. By integrating Speech perception in Noise (BKB-SiN), self-reported hearing ability (SSQ12), and Tinnitus Reduction (TRQ), this work provides a comprehensive evaluation of cochlear implant outcomes.

## Methods

### Participants

Participants in this retrospective cohort study, conducted at a tertiary implant teaching hospital, were adult CI users with bilateral post lingual deafness. To be included in the study, participants had to be fluent in English, be an adult (at least 18-years old at implantation) and have used a CI for at least 12-months. Participants were divided into three groups, depending on their age at implantation: 18–60 years old, 60–75-years-old, and >75 years-old.

### Study design

Demographic data collected included age at implantation, CI experience, years since onset of HL, CI experience, gender, average pre-operative PTA thresholds (500, 1000, 2000 and 4000 Hz) for the Implanted Ear (IE) and the Contralateral Ear (CE) and onset of HL. Each assessment was recorded reoperatively and at three, six, and 12-months of CI use.

### Functional measures

#### Speech understanding in quiet

Speech understanding in quiet was assessed via the Consonant-Nucleus-Consonant (CNC) word test.^23^ A single speaker setup in free field was used with the speaker placed 1 metre away at 0 ° azimuth. Each word was presented at 65 dB SPL. If the contralateral ear had a 3-Frequency Average Hearing Loss (3FAHL) of <65 dB SPL, appropriate masking using wideband noise via an insert headphone was applied. Scores are given in percentage correct (thus, the higher the score, the better the result).

#### Speech understanding in noise

Speech perception in noise was assessed via Bamford-Kowal-Bench Adaptive Speech-in-Noise test (BKB-SiN),^24^ which evaluates the Signal-to-Noise Ratio (dB SNR) needed to achieve a score of 50% of the words correct, thus lower dB SNR indicates better results. Speech and noise were presented through a speaker placed 1 meter directly in front of the participant.

### Patient reported outcome measures

#### Self-perceived hearing ability

Participants’ self-perceived hearing ability was assessed via the short form of the Speech, Spatial and Qualities of Hearing scale (SSQ12).^25^ Twelve questions are answered on an 11-item Likert scale in which 0 is the worst score and 10 is the best score. The Global score is the mean of the answered questions. Higher scores indicate greater self-perceived hearing ability.

#### Tinnitus reduction

Tinnitus reduction was assessed via the Tinnitus Reaction Questionnaire (TRQ).^26^ This was completed only by participants who reported having tinnitus. The TRQ consists of 26 questions answerable on a scale of 0–4, with a possible total score ranging from 0–104. These responses are summed to give an indication of the impact tinnitus has on an individual’s wellbeing.^27^ Lower scores indicate a reduction in tinnitus.

### Statistical analysis

Statistical analyses were conducted using SPSS Statistics V25 (IBM, Armonk, New York, US). Statistical significance was set to p ≤ 0.05. For multiple comparisons p-values were adjusted applying the Holm-Sidak correction method to avoid the type I error. The Shapiro Wilk test and graphical examination confirmed non-normal distribution of the data. Participants were divided into three groups based on their age at implantation: younger CI users (18 – <60 years), older CI users (60 – <75 years) and elderly CI users (>75-years). All statistical analyses were performed for the whole group and for the age stratified groups.

The Friedman test was applied to check for a significant improvement over all tested intervals. If significant, post-hoc pairwise comparisons using the Wilcoxon signed-rank test were performed.

The Mann-Whitney *U*-test was conducted to examine a significant difference between the three age groups at all postoperative (postOp) test intervals.

To look at the relationship of individual independent variables on outcome improvement (i.e., difference between preoperative (preOp) and 12-months postOp testing), simple linear regression models for metric variables, and univariate ANOVA models for categorical variables were applied. As a next step, in a stepwise forward approach the influence of all variables on the individual outcome improvement (i.e., difference between preOp and 12-months postOp testing) was examined with univariate ANOVA models. Variables like age at implantation, preOp Pure Tone Audiometry (PTA) thresholds for the implanted ear, preOp PTA thresholds for the contralateral ear, CI experience, years since onset of Hearing Loss (HL), gender, onset of Hearing Loss (HL), and aetiology of Hearing Loss (HL) were included.

## Results

### Participants

242 CI users were included in this study as they met the inclusion criteria and had either a pre-operative test result and one post-operative test result, or at least two post-operative test results per study outcome. 198 CI users were unilaterally implanted, 35 CI users were bilaterally implanted but only one ear was tested, and 9 CI users were tested in both ears. 72 CI users were allocated to the younger group (18 – <60 years), 105 CI users to the older group (60 – <75 years), and 65 CI users to the elderly group (>75-years). Further demographic information is given in Table 1.

**Table 1:** Participant demographics and clinical characteristics.

Gender (n) Male : Female	134 : 108
**Age at implantation (years): mean ± SD (range)**	64.6 ± 15.95 (18.7–89.8 yrs)
**CI experience (years) mean ± SD (range)**	4.6 ± 3.26 (0.3–17.7 yrs)
**Years since onset of HL: mean ± SD (range)**	24.6 ± 20.18 (0.3–87 yrs)
**Preoperative PTA (dB HL): mean ± SD**	
Implanted ear	96.5 ± 17.11
Contralateral ear	73.7 ± 28.21
**Onset (*****n*****)**	
Birth	20 (8.5%)
Gradual	159 (65.2%)
Sudden	63 (26.3%)
**Aetiology *(n)***	
ISSNHL	21 (8.9%)
Meniere's Disease	19 (8.1%)
Genetic	6 (2.5%)
Incomplete Partition	4 (1.7%)
Noise Induced Hearing Loss	28 (11.9%)
Middle Ear Pathology	24 (10.2%)
Otosclerosis	16 (6.8%)
Ototoxicity	7 (2.9%)
Neuropathy	6 (2.5%
Meningitis	6 (2.5%
MMR	8 (3.4%)
Presbycusis	28 (11.9%)
Trauma	8 (3.4%)
Vascular	5 (2.1%)
Virus	11 (4.7%)
Other	4 (1.7%)
Unknown	41 (16.9%)

PTA, Pure tone average 250 Hz‒8 kHz; ISSNHL, Idiopathic Sudden Sensorineural Hearing Loss. Aetiology of ‘Other’ includes trauma, autoimmune disease, vascular, ototoxicity, incomplete partitioning, meningitis, or neuropathy. CI experience relates to the time (years) from switch on of the first implant.

### Functional results

#### Speech understanding in quiet (CNC)

The mean and the median (±SD) for CNC in quiet are shown for the whole group and the age groups per test interval in Table 2. A significant improvement over all tested intervals was reached for the whole group (Chi-Square = 97.801; df = 3; *p* < 0.001; n = 57), and for the three age groups (*p* < 0.001 each). Post-hoc performed pairwise comparisons confirmed a significant improvement from preOp to all postOp test results for the whole group and for the age stratified groups (Wilcoxon signed-rank test: *p* < 0.001 each). The improvement was not significant from 3- to 6-months postOp testing for the age stratified groups (*p* = 0.064 to *p* = 0.490), and from 6- to 12-months postOp testing for the whole group (*p* = 0.082), and for the age stratified groups (*p* = 0.211 to *p* = 0.683).

**Table 2:** Mean/median ± SD (standard deviation) for CNC in quiet (% correct), SIN in noise (dB SNR), SSQ12 (higher scores indicate greater self-perceived hearing ability) and TRQ (lower scores indicate reduction in tinnitus).

	PreOp mean/median (±SD) n	3m postOp mean/median (±SD) n	6m postOp mean/median (±SD) n	12 m postOp mean/median (±SD) n
CNC				
Whole group	15.6/8.0 (18.4) n = 205	53.9/55.0 (20.7) n = 131	58.9/61.0 (18.6) n = 145	62.6/66.0 (17.7) n = 157
18 ‒ < 60 yrs	13.3/10.0 (14.9) n = 53	57.4/63.0 (24.2) n = 31	61.6/67.5 (20.7) n = 38	65.0/67.5 (19.8) n = 48
60 ‒ < 75 yrs	15.3/6.5 (18.31) n = 96	53.7/57.5 (21.14) n = 66	59.5/57.5 (18.26) n = 66	63.4/67.5 (15.98) n = 66
> 75 yrs	18.9/9.5 (21.5) n = 52	50.0/51.0 (16.2) n = 31	54.9/57.0 (17.6) n = 37	58.6/63.0 (17.6) n = 42
SIN				
Whole group	10.5/9.0 (7.22) n = 93	10.6/9.7 (5.85) n = 62	9.3/8.5 (5.43) n = 70	9.3/9.0 (5.84) n = 69
18 ‒ < 60 yrs	9.3/7.0 (6.64) n = 24	9.2/8.5 (5.45) n = 15	8.9/8.0 (4.74) n = 19	7.9/6.7 (5.99) n = 22
60 ‒ < 75 yrs	11.5/9.0 (8.00) n = 44	11.4/12.0 (5.70) n = 33	9.3/9.5 (5.49) n = 33	10.9/11.0 (5.57) n = 29
> 75 yrs	10.3/10.5 (6.01) n = 23	10.7/9.2 (6.61) n = 12	9.2/7.5 (6.59) n = 15	9.1/9.5 (5.66) n = 15
SSQ12				
Whole group	2.6/2.4 (1.83) n = 66	4.7/4.9 (1.76) n = 32	4.2/4.3 (1.86) n = 39	4.3/4.3 (1.92) n = 40
18 ‒ < 60 yrs	2.4/2.4 (1.65) n = 22	3.8/3.2 (2.22) n = 8	4.2/4.2 (2.11) n = 13	4.2/4.5 (1.94) n = 15
60 ‒ < 75 yrs	2.0/1.1 (1.85) n = 24	5.2/5.4 (1.61) n = 12	4.2/4.6 (1.84) n = 16	4.4/4.3 (1.99) n = 17
> 75 yrs	3.4/3.1 (1.81) n = 20	4.9/4.8 (1.48) n = 12	4.0/4.1 (1.73) n = 10	4.3/3.6 (2.0) n = 8
TRQ				
Whole group	20.4/6.0 (26.20) n = 73	5.6/0 (14.39) n = 40	5.0/0 (9.29) n = 46	4.9/0 (13.09) n = 50
18 ‒ < 60 yrs	26.7/13.0 (28.7) n = 23	3.1/0 (5.13) n = 9	6.5/0 (10.86) n = 18	4.7/0 (10.61) n = 15
60 ‒ < 75 yrs	24.2/14.0 (27.28) n = 32	10.8/0.5 (20.19) n = 18	7.1/2.5 (9.92) n = 16	4.8/0 (13.85) n = 22
> 75 yrs	5.4/0 (12.79) n = 18	0.08/0 (0.27) n = 13	0/0 (0) n = 12	5.3/0 (15.23) n = 13

No significant difference between the three age groups was found at all postOp test intervals (*p* = 0.060 to *p* = 0.423).

Results of linear regression analyses showed a significant individual effect on CNC improvement for “*PreOp PTA thresholds for the implanted ear*” (*t* = 2.830; *p* = 0.006). No significant individual effect on CNC improvement was found for “*age at implantation*” (*t* = −0.726; *p* = 0.470), “*preOp PTA thresholds for the contralateral ear*” (*t* = 1.095; *p* = 0.276), “*CI experience*” (*t* = −0.766; *p* = 0.445), and “*years since onset of HL*” (*t* = 1.578; *p* = 0.118). In the full univariate ANOVA model “*PreOp PTA thresholds for the implanted ear*”, “y*ears since onset of HL*” (Table 3).

**Table 3:** Full univariate ANOVA model with the dependent variable CNC improvement (difference between preOp and 12-months postOp testing).

Independent variables	*F*	p-values (2-sided)
Age at implantation	*F* (1; 63) = 0.173	0.678
PreOP PTA implanted ear	*F* (1; 63) = 12.830	0.001^a^
PreOP PTA contralateral ear	*F* (1; 63) = 3.962	0.055
CI experience	*F* (1; 63) = 0.012	0.913
Years since onset of HL	*F* (1; 63) = 4.430	0.039^a^
Onset of HL	*F* (2; 63) = 2.845	0.066

a Significant effect on CNC improvement.

#### Speech understanding noise (BKB-SiN)

The mean and the median (±SD) for BKB-SIN in noise for the whole group and the age groups per test interval are shown in Table 2. BKB-SIN in noise performance did not change significantly over all tested intervals for the whole group (Chi-Square = 0.853; df = 3; *p* = 0.837; n = 10), and for the three age groups (*p* = 0.500 to *p* = 0.919; n = 2 to n = 5).

No significant difference between the three age groups was found at all postOp test intervals (*p* = 0.080 to *p* = 0.972).

Results of linear regression analyses showed a significant effect on BKB-SIN improvement for “*CI experience*” (*t* = 2.276; *p* = 0.028). No significant individual effect on BKB-SIN improvement was found for “*age at implantation*” (*t* = −1.421; *p* = 0.162), “*preOp PTA thresholds for the implanted ear*” (*t* = 1.496; *p* = 0.144), “*preOp PTA thresholds for the contralateral ear*” (*t* = 1.301; *p* = 0.203), and “*years since onset of HL*” (*t* = 1.418; *p* = 0.164). In the full univariate ANOVA model no significant effect on BKB-SIN improvement was found (Table 4).

**Table 4:** Full univariate ANOVA model with the dependent variable BKB-SIN improvement (difference between preOp and 12-months postOp testing).

Independent variables	*F*	p-values (2-sided)
Age at implantation	*F* (1; 7) = 2.040	0.196
PreOP PTA implanted ear	*F* (1; 7) = 0.208	0.662
PreOP PTA contralateral ear	*F* (1; 7) = 0.830	0.392
CI experience	*F* (1; 7) = 0.058	0.817
Years since onset of HL	*F* (1; 7) = 0.183	0.682
Onset of HL	*F* (1; 7) = 0.381	0.557

### Patient reported outcome measures

#### Self-perceived hearing ability (SSQ12)

The mean and the median (±SD) of the SSQ12 are shown for the whole group and the age stratified groups per test interval in Table 2. A significant improvement over all tested intervals was found for the whole group (Friedman test: Chi-Square = 12.200; df = 3; *p* = 0.007; n = 6). Significant improvement from preOp to all postOp test results for the whole group (Wilcoxon signed-rank test: *p* < 0.001), and for the older age group (*p* = 0.001 to *p* = 0.004). For the younger age group, the improvement was significant from preOp to 12-month postOp testing (*p* = 0.006).

There was no significant difference in self-perceived hearing ability between the three age groups at all postOp tested intervals (*p* = 0.189 to *p* = 1.00).

Results of linear regression analyses did not show a significant individual effect on improvement in self-perceived hearing ability for “*age at implantation*” (t = −0.300; *p* = 0.766), “*preOp PTA thresholds for the implanted ear*” (t = −0.066; *p* = 0.948), “*preOp PTA thresholds for the contralateral ear*” (*t* = 0.332; *p* = 0.742), “*CI experience*” (*t* = −1.443; *p* = 0.159), and “*years since onset of HL*” (*t* = 0.713; *p* = 0.481). Also no individual significant effect of “*gender*” (*F* (1; 31) = 0.056; *p* = 0.814) and “*onset of HL*” (*F* (2; 30) = 0.397; *p* = 0.676) was reached. In the full univariate ANOVA model “*CI experience*” was significant (see Table 5).

**Table 5:** Full univariate ANOVA model with the dependent variable SSQ12 (difference between preOp and 12-months postOp testing).

Independent variables	*F*	p-values (2-sided)
Age at implantation	*F* (1; 3) = 2.451	0.215
PreOP PTA implanted ear	*F* (1; 3) = 3.845	0.145
PreOP PTA contralateral ear	*F* (1; 3) = 0.691	0.467
CI experience	*F* (1; 3) = 11.447	0.043^a^
Years since onset of HL	*F* (1; 3) = 3.581	0.155
Onset of HL	*F* (1; 3) = 1.645	0.290

a Significant effect on SSQ12 improvement.

#### Tinnitus reduction (TRQ)

The mean and the median (±SD) TRQ results for the whole group and the age groups per test interval are shown in Table 2.

Tinnitus perception did not differ significantly between the three age groups at all postOp test intervals (*p* = 0.127 to *p* = 1.000).

Results of linear regression analyses showed a significant effect of “*age at implantation*” on tinnitus reduction (*t* = −3.067; *p* = 0.004). No significant individual effect on tinnitus reduction was found for “*preOp PTA thresholds for the implanted ear*” (*t* = 0.618; *p* = 0.540), “*preOp PTA thresholds for the contralateral ear*” (*t* = 1.700; *p* = 0.097), “*CI experience*” (*t* = 1.811; *p* = 0.077), and “*years since onset of HL*” (*t* = −0.473; *p* = 0.638). Also no significant individual effect of “*gender*” (*F* (1; 41) = 0.000; *p* = 0.993), and “*onset of HL*” (*F*(2; 39) = 0.392; *p* = 0.678), was reached. “*Age at implantation*” remained significant in the full univariate ANOVA model (Table 6).

**Table 6:** Full univariate ANOVA model with the dependent variable Tinnitus reduction (difference between preOp and 12-months postOp testing).

Independent variables	*F*	p-values (2-sided)
Age at implantation	*F* (1; 12) = 6.355	0.027^a^
PreOP PTA implanted ear	*F* (1; 12) = 0.330	0.576
PreOP PTA contralateral ear	*F* (1; 12) = 0.600	0.454
CI experience	*F* (1; 12) = 1.061	0.323
Years since onset of HL	*F* (1; 12) = 0.876	0.368
Onset of HL	*F* (1; 12) = 0.402	0.538

a Significant effect on TRQ improvement.

## Discussion

As life expectancy increases, so too does the number of people who have a severe to profound hearing loss. Many of these people could potentially benefit from using a CI. Appropriate treatment of hearing loss is a key factor in maintaining independence, as well as reducing a significant modifiable risk factor for cognitive decline.^28^ Cosetti et al. (2017) showed a complex link between cognitive testing and speech perception, indicating that CI use slows age-related cognitive decline.^29^ Early intervention for hearing loss may also reduce the trajectory of cognitive decline, indicating that appropriate hearing rehabilitation is imperative.^30^ Age is thought to be one barrier to cochlear implantation, with elderly patients or family members reporting they are “too old” for CI.^31^

### Functional outcomes

Speech understanding in quiet scores increased across all age groups from pre- to post-operatively, with speech scores increasing over the first 12-months in all three groups. No significant difference was observed between the different groups. The greatest improvement in speech understanding scores was in the first three months of CI use in all three groups. This is consistent with Amin et al. (2020), who also observed the largest improvement in speech understanding during the first three months post-implantation. Further, Amin et al. (2020) found no difference in speech understanding outcomes when splitting the cohort into two groups based on age (70–79 years and >80 years).^32^ Wong et al. (2015) similarly found no significant correlation between age and speech outcomes when examining 150 participants implanted after the age of 75 years.^9^ Given that our cohort was divided into three age groups, it appears that age did not significantly impact the speech understanding outcomes in quiet.

Understanding speech in noise requires greater listening effort than understanding speech in quiet because the brain needs to filter out the extraneous noise, thereby making it more cognitively taxing.^33^ While younger users might be expected to perform better in noise tasks compared with older users, we found no significant difference in BKB-SiN scores between the age groups. This finding is consistent with previous studies.^18^, ^32^ However, unlike Amin et al.’s (2021) who found a significant improvement in BKB-SiN from pre- to postoperative when using a fixed SNR of 10 dB, we did not observed significant improvement over time. Understanding speech in noise benefits from binaural cues to help segregate speech and noise, but our SiN configuration (with speech and noise from the same speaker 1 metre directly in front of the participant) excludes this possibility, providing cues that primarily facilitate binaural summation. In normal hearing, binaural summation accounts for an improvement of 6 dB at normal conversation level. Given that our cohort had a significant hearing loss in the contralateral ear (albeit aided) (Table 1), the SiN setup used here may have been simply too challenging for these listeners, even with CI provision. Additionally, we used an adaptive speech in noise testing which is much more challenge than a fixed SNR presentation. While this configuration likely limited performance, it is encouraging that CI provision does not impede speech understanding in noise.

Age at implantation alone may not be a reliable prognostic factor in CI outcomes. Elderly people generally have longer durations of deafness than younger CI recipients, which complicates efforts to isolate the effect of age. A further challenge lies in identifying the precise onset of hearing loss based on patient recall, which is prone to recall bias. It has previously been shown that, in CI users, duration of deafness and pre-operative speech understanding scores are more closely correlated with post-operative outcomes than they are with age at implantation.^17^, ^34^ which aligns with our findings.

### Patient reported outcome measures

SSQ12 results support previous findings that demonstrated significant improvement in self-perceived hearing ability over time.^35^ While there was no pre-operative difference in SSQ12 scores between the age groups, the < 60 years group had a significantly a smaller improvement at three months compared with the other groups. This difference resolved over time, with no significant differences present at 6- or 12-months. Given that speech perception scores in quiet and in noise were similar across all groups at three months, the lower SSQ12 scores in the <60-years group may reflect higher pre-operative expectations or more immediate adjustment challenges compared to older adults, who might have approached the initial stages of rehabilitation with lower expectations.

TRQ scores declined significantly from pre- to post-operatively, with the largest reduction occurring within the first three months of CI use. CI experience continued to influence TRQ scores, with participants experiencing further tinnitus reduction throughout the 12-month observation period. This effect was consistent across all age groups, indicating that CI use significantly alleviates tinnitus-related distress regardless of age. These findings align with prior research documenting the robust effect of CIs on tinnitus reduction, independent of recipient’s age.

The findings presented here bolster the message that there is no upper age limit for cochlear implantation. Older individuals benefit from implantation, and often to the same degree as their younger peers. This message should be considered in professional, community and family education and outreach programs. Likewise, hearing surveillance, therapy, and rehabilitation services should be integrated into elderly care models. Individuals with severe/profound hearing loss should not be excluded from the appropriate treatment avenue on the basis of age alone.

### Limitations

Given the retrospective nature of this study, many of our participants were analysed when our standardised protocol was still in development, accounting for the missing data in BKB-SiN and SSQ12 assessments. We limited this study to 12-months of CI use to maintain the statistical strength when comparing groups; future studies would benefit from investigating the longer-term impact that age has on CI outcomes and the potential influence of cognitive decline.

## Conclusion

Each age group of adults had increased self-reported hearing abilities (a subjective measure) and speech understanding scores (an objective measure) after 12-months of CI use. Intergroup comparison showed that age at implantation did not significantly affect scores. We therefore conclude that elderly people (> 75-years) are acceptable as candidates for a CI and should be offered the same opportunity to benefit from CI use as other adult candidates.

## Ethics

Ethics approval was obtained from the South Metropolitan Health Ethics Committee (reference number: RGS0000005687) Subjects have given their written informed consent to participate in this study.

## Funding

Dayse Tavora-Vieira holds a research fellowship grant from 10.13039/501100001063Raine Medical Research Foundation.

## Declaration of competing interest

The authors declare no conflicts of interest.
